# Against the Odds: Safe Intubation in a Young Patient With a Massive Tonsillar Tumor

**DOI:** 10.7759/cureus.85307

**Published:** 2025-06-03

**Authors:** Ahmad Nabil, Khaled S Abuamra, Hatem Ibrahim, Sherin Abdelhamid, Cornelia Weidinger

**Affiliations:** 1 Department of Anesthesia and Intensive Care, Ain Shams University, Cairo, EGY; 2 Department of Anesthesia, Dubai Hospital, Dubai Health, Dubai, ARE

**Keywords:** airway management, nasogastric tube, nasotracheal intubation, oropharyngeal tumor, video laryngoscope

## Abstract

Oropharyngeal tumors pose significant challenges for airway management and nasogastric tube (NGT) insertion due to anatomical distortion. We report a case of a young male who underwent excision of a large left oropharyngeal mass, diagnosed as a pleomorphic adenoma. The case required meticulous planning for nasotracheal intubation and preprocedural NGT insertion, as requested by the surgeon, due to tumor-related airway distortion. After induction of general anesthesia, right-sided nasotracheal intubation was performed uneventfully using the video laryngoscope. Left-sided NGT insertion was completed preprocedurally without complications. This report highlights the strategic use of video laryngoscopy, preoperative coordination, and tailored NGT placement in managing complex head and neck cases, ensuring safe airway and nutritional access.

## Introduction

Oropharyngeal tumors complicate airway management by distorting anatomy and increasing the risk of obstruction [1]. These tumors can obstruct the airway, alter normal anatomical landmarks, and necessitate advanced techniques for securing the airway safely. The video laryngoscope has emerged as a critical tool in such cases, enhancing visualization of the glottis and improving intubation success rates in challenging airways [2]. Additionally, preprocedural nasogastric tube (NGT) insertion in patients with oropharyngeal tumors requires careful planning to avoid trauma, misplacement, or exacerbation of airway compromise. The anatomical distortion caused by the tumor may limit nasal access, requiring strategic selection of the insertion side and technique. We present a case of a 27-year-old male patient with a large oropharyngeal tumor, emphasizing the anesthetic challenges of nasotracheal intubation and preprocedural NGT insertion, and the strategies that ensured a successful outcome.

## Case presentation

A 27-year-old male with no significant past medical history presented for excision of a left oropharyngeal mass. Pre-operative imaging from a CT scan of the neck (with and without contrast) revealed a well-defined, oval-shaped, heterogeneous mass (4.5 x 3.5 x 3 cm) centered at the left tonsillar fossa, isointense on non-contrast CT with heterogeneous enhancement post-contrast, displacing the tongue base, genioglossus, and medial pterygoid muscles anteriorly without infiltration.

The mass partially obstructed the left nostril, complicating airway and NGT planning. The patient’s American Society of Anesthesiologists (ASA) Physical Status was 2 and STOP-Bang score was 3, and his Mallampati score was II, indicating a moderate risk of difficult intubation. The patient did not report dysphagia or voice changes, and no stridor or dyspnea was noted preoperatively.

**Figure 1:**
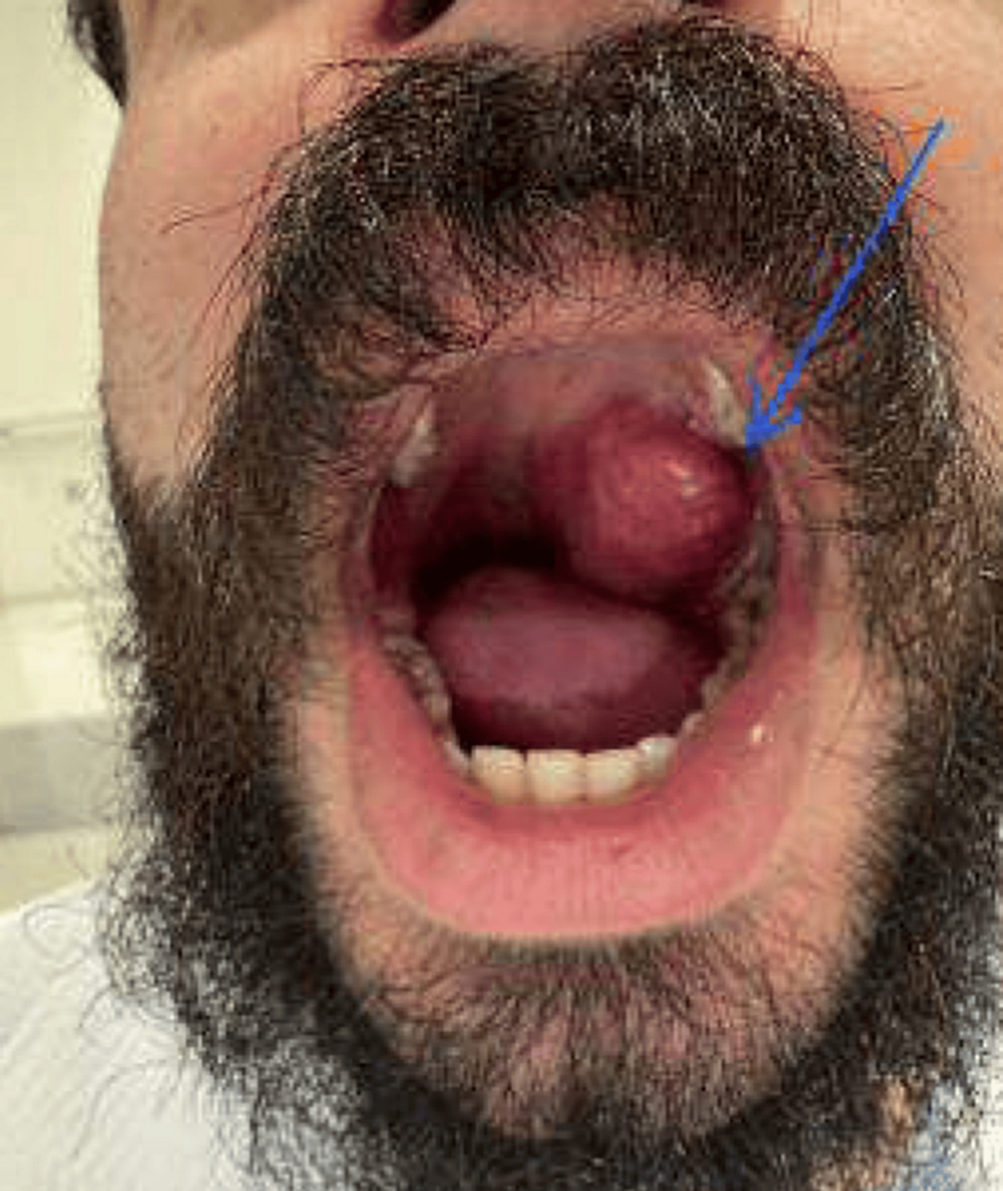
Left tonsillar mass

During a preprocedural fiberoptic laryngoscopy by the surgeon, the nasal examination revealed septal deviation and mucosal edema without signs of deformity, injury, or congestion. Both nostrils were clear, showing no foreign bodies, epistaxis, septal hematomas, or occlusions. The turbinates bilaterally appeared enlarged, swollen, and pale. The oral and throat assessment showed pink, lesion-free lips, moist mucous membranes, normal dentition without tenderness or abnormalities, and a midline tongue without deviation. The palate was free from masses or lesions, while the oropharynx was clear, with a midline uvula and no swelling, erythema, or exudate. The tonsils showed no exudate or abscesses. Notably, a well-defined, non-infiltrating left-sided soft palate tumor (3x3 cm) was present, displacing the uvula to the opposite side, but it remained encapsulated, non-ulcerated, and without signs of bleeding or discharge (Figure 1).

Preoperative assessment and planning

The anesthesia team conducted a thorough preoperative evaluation, noting a history of childhood tonsillectomy and heavy smoking, increasing airway complication risks. Airway assessment revealed normal mouth opening, full neck range of motion, and a thyromental distance greater than three fingerbreadths. The left-sided mass necessitated right-sided nasotracheal intubation, with the left nostril designated for preprocedural NGT insertion per the surgeon’s request. Following multidisciplinary discussion with the otolaryngology team, nasotracheal intubation using the C-MAC® video laryngoscope (Karl Storz, Tuttlingen, Germany) under general anesthesia was planned, with tracheostomy standby for potential airway obstruction. The patient adhered to nil per os (NPO) status. Laboratory results confirmed normal renal function (serum creatinine 0.72 mg/dL), CBC (14.8 g/dL), and coagulation (INR 1.09), supporting procedural safety (Table 1).

**Table 1:** Laboratory blood investigations

Component	Result	Reference range & units
Hemoglobin	14.8	13.0 - 17.0 g/dL
Creatinine blood	0.72	0.70 - 1.20 mg/dL
International normalised ratio (INR)	1.09	0.8 - 1.1

Anesthetic management and NGT insertion

The patient was prepared in the operating theater. Topical anesthesia was applied to the right nasal passage using 2% lidocaine jelly. Glycopyrrolate (0.2 mg IV) reduced secretions, and midazolam (2 mg IV) provided anxiolysis. General anesthesia was induced with propofol (2 mg/kg) and fentanyl (2 µg/kg), followed by rocuronium (0.6 mg/kg) for neuromuscular blockade where Sugammadex (16 mg/kg, 1,120 mg for our 70 kg patient, 6 vials) was available, with one anesthesia technician assigned to prepare it if needed. Right-sided nasotracheal intubation was performed using the C-MAC video laryngoscope with a Macintosh blade (size 4) and a 6.5 mm cuffed Ring-Adair-Elwyn (RAE) north tube. The intubation was uneventful, with the tube placed at 24 cm from the nares, confirmed by capnography, auscultation, and a full glottic view. Awake fiberoptic intubation was considered but deferred given the patient’s low predicted difficulty score and the anticipated comfort and success with video laryngoscopy. After induction of general anesthesia, left-sided NGT insertion was performed as requested by the surgeon. A 16 Fr NGT was inserted through the left nostril using lubrication and gentle manipulation under video laryngoscopy to ensure accurate placement and avoid tumor trauma. The procedure was uneventful, with placement confirmed by gastric content aspiration and a subsequent chest X-ray. Anesthesia was maintained with desflurane, and the surgical excision proceeded without complications. Histopathology confirmed a 48 mm pleomorphic adenoma, fully excised.

Postoperative care

The patient was transferred to the surgical intensive care unit (SICU) on mechanical ventilation with sedation. A Foley catheter monitored urine output, and antimicrobials, gastrointestinal, and deep vein thrombosis prophylaxis were continued. The patient was weaned and extubated successfully on postoperative Day 1, with no airway or NGT-related complications. The NGT supported early enteral nutrition effectively.

## Discussion

This case highlights the complexities of airway management and NGT insertion in the presence of a large oropharyngeal tumor, which distorted the airway anatomy and posed significant procedural challenges. The use of the C-MAC video laryngoscope was instrumental in achieving successful nasotracheal intubation. Video laryngoscopy provided superior visualization of the glottis, overcoming the anatomical distortion caused by the tumor and the partial obstruction of the left nostril [2]. Imaging from a CECT scan of the neck (with and without contrast) revealed a well-defined, oval-shaped, heterogeneous mass (4.5 x 3.5 x 3 cm) centered at the left tonsillar fossa, isointense on non-contrast CT with heterogeneous enhancement post-contrast, displacing the tongue base, genioglossus, and medial pterygoid muscles anteriorly without infiltration. The lesion indented the uvula to the right, effaced the left oropharynx, and compressed the left eustachian tube, reaching the naso-oropharyngeal junction without bony involvement. Notably, the tumor was non-vascular, and no bleeding was encountered during the procedure, reducing intraoperative risks. 

Recent advancements in video laryngoscopy, such as high-definition imaging and angulated blades, have further improved intubation success rates in complex airways, with studies reporting first-pass success rates exceeding 90% in difficult airway scenarios [3]. The reliability of the C-MAC system was critical in this case, reducing the need for awake fiberoptic intubation, a more invasive and patient-discomforting approach [4]. The C-MAC video laryngoscope has been studied as an alternative to fiberoptic bronchoscopy (FOB) for difficult airway management, particularly in cases requiring nasotracheal intubation. 

A randomized comparative study found that awake intubation using the C-MAC video stylet was easier and more successful than FOB, with a higher first-attempt success rate (90% vs. 72.5%) and shorter intubation time. Another study evaluating C-MAC D-BLADE-guided videolaryngoscopy versus FOB for nasotracheal intubation in oropharyngeal carcinoma patients reported that C-MAC had a significantly shorter intubation time and improved visualization [5]. A comparative analysis of C-MAC versus flexible fiberoptic scope highlighted that video laryngoscopy provides indirect visualization, reducing the need for direct laryngeal exposure while maintaining effective airway control. While FOB remains the gold standard for complex airway cases, C-MAC has demonstrated comparable efficacy in scenarios with anatomical distortion or limited direct visualization, as seen in this case with a wide mouth opening facilitating the procedure [6].

Preprocedural NGT insertion through the left nostril, as requested by the surgeon, was a critical component of the perioperative plan to ensure postoperative nutritional support without compromising the surgical field. The use of video laryngoscopy during NGT placement minimized the risk of trauma to the tumor or misplacement, which is particularly important in anatomically altered airways where blind insertion could lead to complications such as bleeding or perforation [7]. This case underscores the importance of tailoring NGT insertion to the patient’s anatomy and surgical requirements.

Multidisciplinary preoperative coordination with the otolaryngology team was pivotal in aligning the airway and NGT strategies, optimizing procedural safety, and ensuring clear communication of surgical goals [1]. The availability of tracheostomy standby adhered to airway management guidelines and provided a safety net for potential airway obstruction, a critical consideration given the tumor’s size and location [2]. Furthermore, the patient’s history of heavy smoking increased the risk of airway complications, such as laryngeal edema or bronchospasm, which was mitigated through preoperative optimization (e.g., glycopyrrolate to reduce secretions) and careful anesthetic management. Sugammadex (16 mg/kg, 1,120 mg for a 70 kg patient, 6 vials) was available, with one anesthesia technician assigned to prepare it if needed, ensuring rapid reversal of neuromuscular blockade if required.

The case also highlights the broader implications of advanced visualization tools in modern anesthesia practice. Video laryngoscopy not only facilitates intubation but also enhances training and documentation, allowing anesthesiologists to review and refine techniques for complex cases [3]. However, challenges remain, including the cost and availability of advanced equipment in resource-limited settings, which may limit widespread adoption. Future research should focus on cost-effective visualization tools and standardized protocols for NGT insertion in head and neck surgery to further improve patient outcomes.

In summary, this case demonstrates the efficacy of video laryngoscopy and tailored NGT placement in managing a challenging airway. The integration of advanced technology, meticulous preoperative planning, and multidisciplinary collaboration was essential to achieving a safe and successful outcome.

## Conclusions

This case illustrates the successful management of a challenging airway and preprocedural NGT insertion in a patient with an oropharyngeal tumor. The C-MAC®-guided nasotracheal intubation and precise NGT placement under video visualization ensured a safe and effective outcome. Anesthesiologists must integrate advanced visualization tools, preoperative multidisciplinary planning, and tailored techniques to navigate such complexities.

## References

[1] Wong P, Wong J, Mok MU (2016). Anaesthetic management of acute airway obstruction. Singapore Med J.

[2] Apfelbaum JL, Hagberg CA, Connis RT (2022). 2022 American Society of Anesthesiologists practice guidelines for management of the difficult airway. Anesthesiology.

[3] Huang HB, Peng JM, Xu B, Liu GY, Du B (2017). Video laryngoscopy for endotracheal intubation of critically ill adults: a systemic review and meta-analysis. Chest.

[4] Cook TM, Woodall N, Frerk C (2011). Major complications of airway management in the UK: results of the Fourth National Audit Project of the Royal College of Anaesthetists and the Difficult Airway Society. Part 1: anaesthesia. Br J Anaesth.

[5] Kumar A, Gupta N, Bhargava T (2024). A comparative evaluation of fibreoptic bronchoscopy versus C-MAC(®) D-BLADE-guided videolaryngoscopy for nasotracheal intubation under general anesthesia in oropharyngeal carcinoma surgery patients. Can J Anaesth.

[6] Yumul R, Elvir-Lazo OL, White PF (2016). Comparison of the C-MAC video laryngoscope to a flexible fiberoptic scope for intubation with cervical spine immobilization. J Clin Anesth.

[7] Ahmad A, Abdul-Hamid A, Abdul-Hamid A (2015). Challenging nasogastric tube insertion made easy. Ann R Coll Surg Engl.

